# Effects of biochar and vermicompost on growth and economic benefits of continuous cropping pepper at karst yellow soil region in Southwest China

**DOI:** 10.3389/fpls.2023.1238663

**Published:** 2023-09-20

**Authors:** Meng Zhang, Yanling Liu, Quanquan Wei, Lingling Liu, Xiaofeng Gu, Jiulan Gou, Ming Wang

**Affiliations:** ^1^ Institute of Soil and Fertilizer, Guizhou Academy of Agricultural Sciences, Guiyang, China; ^2^ College of Eco-Environmental Engineering, Guizhou Minzu University, Guiyang, China

**Keywords:** biochar, vermicompost, continuous cropping pepper, yield and quality, fertilizer utilization, economic benefits

## Abstract

Recently, biochar (B) and vermicompost (V) have been widely used as amendments to improve crop productivity and soil quality. However, the ameliorative effects of biochar and vermicompost on the continuous cropping of pepper under open-air conditions, particularly in the karst areas of southwestern China, remain unclear. A field experiment was conducted to study the effects of biochar and vermicompost application, alone or in combination, on the yield, quality, nutrient accumulation, fertilizer utilization, and economic benefits of continuous pepper cropping from 2021 to 2022. The experiment included six treatments: CK (no fertilizer), TF (traditional fertilization of local farmers), TFB (TF combined with biochar of 3000 kg·ha^-1^), TFV (TF combined with vermicompost of 3000 kg·ha^-1^), TFBV1 (TF combined with biochar of 1500 kg·ha^-1^ and vermicompost of 1500 kg·ha^-1^), and TFBV2 (TF combined with biochar of 3000 kg·ha^-1^ and vermicompost of 3000 kg·ha^-1^). Compared with the TF treatment, biochar and vermicompost application alone or in combination increased the yield of fresh pod pepper by 24.38–50.03% and 31.61–88.92% in 2021 and 2022, respectively, whereas the yield of dry pod pepper increased by 14.69–40.63% and 21.44–73.29% in 2021 and 2022, respectively. The application of biochar and vermicompost reduced the nitrate content and increased the vitamin C (VC) and soluble sugar content of the fruits, which is beneficial for improving their quality. Biochar and vermicompost application alone or in combination not only increased nutrient uptake but also significantly improved agronomic efficiency (AE) and recovery efficiency (RE). In addition, although the application of biochar or vermicompost increased production costs, the increase in yield improved net income (ranging from 0.77 to 22.34% in 2021 and 8.82 to 59.96% in 2022), particularly in the TFBV2 treatment. In conclusion, the use of biochar and vermicompost amendments had a positive effect on the productivity and economic benefits of continuous pepper cropping, and the co-application of biochar and vermicompost could be an effective nutrient management strategy for the continuous cropping of pepper in the karst mountain areas of southwest China.

## 1 Introduction

Modern agriculture is facing new challenges owing to climate change, environmental degradation, and population growth pressure. The growing demand for agricultural products further increases the pressure on crop production and necessitates agricultural activities to minimize its negative impact on the environment (Bommarco et al., 2013). Therefore, improving the sustainable productivity of existing farmlands is the key to meeting future global crop demands with minimal environmental impacts. The monoculture intensification of cash crops, such as vegetables and fruit trees, has led to continuous cropping obstacles and replanting diseases, which have significantly affected the productivity of arable land. This poses a serious threat to regional food security and environmental safety (Tilman et al., 2011). Many studies have confirmed that the dysregulation of soil microbial diversity is the underlying cause of continuous cropping obstacles (Fan et al., 2022; Gu et al., 2022). Moreover, long-term continuous cropping of crops will not only inhibit the growth and reproduction of beneficial microorganisms and promote the accumulation of plant pathogens but also lead to a decrease in crop yield and quality (Li et al., 2022). At present, organic remediation materials have received much attention as widely used soil amendments because of their beneficial effects in enhancing crop yield and quality and improving soil quality. Many studies have confirmed that organic remediation materials can overcome or mitigate the barriers to continuous cropping by increasing organic carbon sources and soil microbial diversity (Su et al., 2022; Zhang et al., 2022a; Zhang et al., 2022b).

Biochar is an insoluble, stable, highly aromatic, carbon-rich solid substance produced by the slow pyrolysis of biological residues at high temperatures (<700 °C) under hypoxic conditions (Hossain et al., 2020; He et al., 2021). Biochar can not only improve soil physicochemical properties, such as increasing pH, porosity, and water retention, and reducing soil bulk density but also improve soil fertility and effectively restore degraded soil owing to its rich nutrient elements (Yang et al., 2019; Liu et al., 2021). In addition, biochar can affect the diversity of microbial communities by reducing the number of harmful fungi in the soil, which can help prevent the reproduction and growth of harmful plant pathogens (Zhang et al., 2021b; Zhang et al., 2021c). Similar studies have also been conducted on vermicompost application. Vermicompost is a natural biological organic fertilizer formed by earthworms using decomposed organic wastes (livestock manure, sludge, straw, etc.) as bait and is excreted after digestion (Atiyeh et al., 2002; Guerrero, 2010). Vermicompost has a large surface area that provides microsites for the microbial decomposition of organic matter and has a strong capacity for nutrient adsorption and retention, as well as an excellent water-holding capacity (Zaller, 2007; Zucco et al., 2015; Haribhushan et al., 2016). In addition, vermicompost has a passivation effect on heavy metal ions in the soil, which can reduce toxicity to plants by reducing the bioavailability and mobility of heavy metal ions (Khan et al., 2019; Shen et al., 2022). Vermicompost is rich in microorganisms that can improve the disease resistance of plants by forming antagonistic or inhibitory effects against many pathogenic bacteria (Zhao et al., 2017; Benazzouk et al., 2020). Currently, biochar and vermicompost are widely used in agricultural production as important organic amendment materials and have shown significant advantages in increasing soil fertility, enhancing crop yield, and improving the quality of agricultural products (Hossain et al., 2020; Tikoria et al., 2022).

China is a major producer of pepper in the world; however, the phenomenon of continuous cropping obstacles in peppers has become a common problem in planting areas, owing to unreasonable cultivation and management measures (Zou et al., 2020; Zou and Zou, 2021). Long-term continuous cropping of pepper has led to the frequent occurrence of pests and diseases, which have caused a continuous decline in pepper yield, quality, and economic benefits, seriously restricting the healthy and sustainable development of pepper (Zhang et al., 2021a; Zhang et al., 2022c). Although many field experiments have confirmed the positive effects of biochar or vermicompost application alone on increasing crop yield and improving soil quality, studies on the effects of biochar and vermicompost application alone or in combination on the biological characteristics, fertilizer utilization, and economic benefits of continuous pepper cropping in karst areas are still unknown. To investigate whether biochar and vermicompost applied alone or in combination have an ameliorative effect on the continuous cropping of pepper in the karst region of southwest China, we conducted a 2-year field experiment to assess their potential for the productivity of continuous cropping pepper.

## 2 Materials and methods

### 2.1 Site description

**Figure 1:**
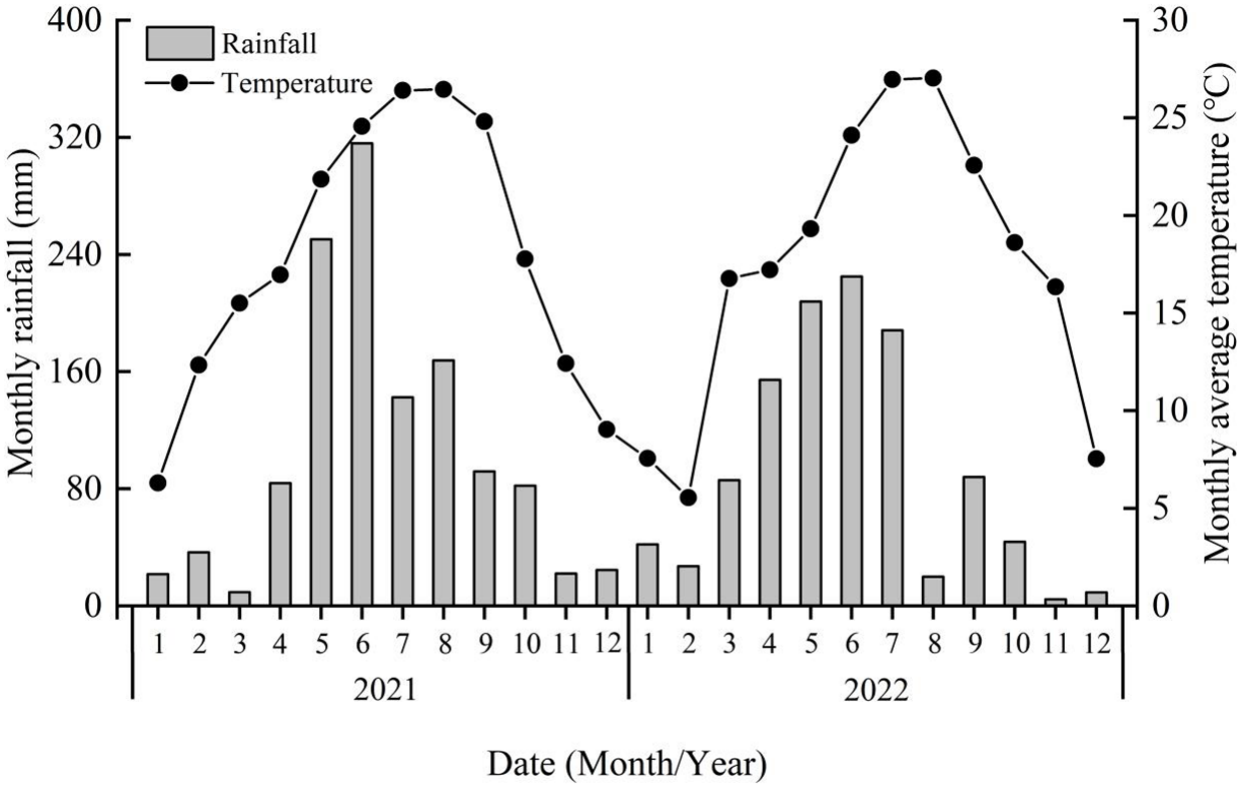
The weather conditions of the experimental site from 2021 to 2022.

The field experiment was conducted in Xinmin Town (27°22’59’’ N, 106°56’14’’ E) from 2021 to 2022 in the Bozhou District, Zunyi City, Guizhou Province, China. Before conducting the experiment, the field was planted with pod pepper for five years from 2016 to 2020. This region has a subtropical monsoon climate, with an average annual rainfall of 891 mm and an average annual temperature of 14.3°C. The weather conditions of the experimental site from 2021 to 2022 were shown in **Figure 1**, where the growth period of peppers was from April to October. The soil type of the experiment region is yellow soil, which is the zonal soil, with a high aluminization intensity, formed under perennial, humid, and bioclimatic conditions in the subtropical zone. Due to the intense leaching caused by the perennial humidity, the exchangeable base content is only 20%, and therefore the base is extremely unsaturated. The basic physicochemical properties of the soil were as follows: pH of 6.19, organic carbon (OC) of 11.88 g·kg^-1^, total nitrogen (TN) of 1.36 g·kg^-1^, total phosphorus (TP) of 1.02 g·kg^-1^, available phosphorus (AP) of 48.60 mg·kg-1, total potassium (TK) of 17.11 g·kg^-1^, available potassium (AK) of 156.83 mg·kg^-1^, electrical conductivity (EC) of 1.42 mS·cm^-1^, and cation exchange capacity (CEC) of 7.06 cmol·kg^-1^.

### 2.2 Experimental material

The pod pepper variety used in the experiment was ‘Zunla 9’, which was bred by the Zunyi Academy of Agricultural Sciences and was the main local planting variety. Chemical fertilizers N-P_2_O_5_-K_2_O 18-6-18, (Guizhou Tianbao Fengyuan Ecological Agricultural Technology Co., Ltd., Xiuwen, China), biochar (Guizhou Institute of Soil and Fertilizer Research, Guiyang, China), and vermicompost (Hubei Tianshenjia Biological Environmental Protection Technology Co., Ltd., Wuxue, China) were used in this experiment. The raw material of biochar was distillers grains which comprise biomass waste generated from the production process of distilled spirits. Biochar was prepared by the oxygen-limited cracking method in a biomass carbonization furnace (SSDP-5000-A, Jiangsu Huaian Huadian Environmental Protection Machinery Manufacturing Co., Ltd.). Briefly, we obtained appropriate amounts of distillers grain samples and put them in the equipment, followed by blowing in N_2_ for 5-10 min to exhaust the excess air in the furnace. The sample was pyrolyzed at 550 °C for 2 h. After cooling, it was passed through a 100-mesh sieve and placed in the shade until subsequent experiments. The structural characteristics of the distillers grain biochar were as follows: specific surface area (SSA) of 2.12 m^2^·g^-1^; single point adsorption total pore volume (SPATPV) of 2.95×10^-3^ m^3^·g^-1^, and average pore size (APS) of 5.55 nm. Vermicompost was obtained by digesting cow manure with earthworms for 60 days and then separating the earthworms from the manure. The nutrient content of biochar and vermicompost were listed in **Table 1**.

**Table 1:** The nutrient content of biochar and vermicompost.

	pH	OC (g·kg^-1^)	TN (g·kg^-1^)	TP (g·kg^-1^)	AP (mg·kg^-1^)	TK (g·kg^-1^)	AK (mg·kg^-1^)
Biochar	8.23	354.86	39.84	9.23	275.47	19.88	748.55
Vermicompost	7.28	113.48	21.57	3.15	485.36	4.95	243.54

OC, stands for organic carbon; TN, stands for total nitrogen; TP, stands for total phosphorus; AP, stands for available phosphorus; TK, stands for total potassium; AK, stands for available potassium.

### 2.3 Experimental design

Field experiments were conducted from 2021-2022 including two pod pepper planting seasons. There were six treatments with three replicates: (1) CK, no fertilizer; (2) TF, traditional fertilization of local farmers, 1500 kg·ha^-1^ of chemical fertilizer; (3) TFB, TF combined with 3000 kg·ha^-1^ of biochar; (4) TFV, TF combined with 3000 kg·ha^-1^ of vermicompost; (5) TFBV1, TF combined with 1500 kg·ha^-1^ of biochar and 1500 kg·ha^-1^ of vermicompost; and (6) TFBV2, TF combined with 3000 kg·ha^-1^ of biochar and 3000 kg·ha^-1^ of vermicompost. It should be noted that the application rates of biochar and vermicompost were determined based on the recommended organic fertilizer application rates by the local agricultural department, and also taking into account the affordability of the farmers. In the experiment, chemical fertilizer, biochar, and vermicompost were applied to the soil as a base fertilizer before transplanting of the pod pepper, and then a rotary tiller was used to mix the fertilizers and the soil evenly. Pod pepper seedlings were transplanted 15 days after ridging and covered with a plastic film. The planting density was 45000 plants·ha^-1^. Each treatment was conducted in a random block and the plot area was 40.50 m^2^ (9.0 m × 4.5 m). In addition, all field management practices were consistent to ensure the accuracy of test results.

### 2.4 Soil sampling and analysis

Before the fertilizers were spread, soil samples (0–20 cm depth) were collected using a soil auger from 15 randomly selected sites. All the soil samples were mixed into one composite sample, and then air-dried, ground, and passed through 1.00 mm and 0.15 mm sieves to measure pH, soil organic matter (SOM), total nitrogen (TN), available phosphorus (AP), and available potassium (AK) (Ning et al., 2022). Soil samples (0-20 cm) were collected at five randomly selected points in each experimental plot using a soil auger after pepper harvest in 2022, and then one composite soil sample was composed. Finally, a total of 18 composite soil samples were obtained. These 18 soil samples were assayed for pH, SOM, TN, AP, and AK according to the methods described above.

### 2.5 Plant sampling and analysis

Six plants were sampled from each plot during the ripening stages of the pod pepper. The fresh pod pepper plants were divided into three parts: stems, leaves, and fruits. It should be noted here that due to the root and stem were connected and both were highly lignified, so that we counted the root and stem as one part and referred to them collectively as the stem. The plant samples were baked first at 105 °C for 0.5 hours and then baked to a constant weight at 60 °C. The dried stems, leaves, and fruits were weighed and summed to obtain the aboveground dry biomass of pod peppers. All dried samples were ground, passed through a 0.25 mm sieve, and digested with a mixture of concentrated H_2_SO_4_ and H_2_O_2_ to determine N, P, and K concentrations (Zhang et al., 2021a). In addition, fresh pod pepper samples from each plot were collected at the mature stage to determine the free amino acid, reducing sugar, VC, and nitrate contents (Zhang et al., 2021a).

### 2.6 Yield of pod pepper

The yield of fresh pod pepper in each plot was weighed according to the maturity of the pod pepper in each plot. The final yield of fresh pepper was calculated according to the weight of multiple harvests. In addition, the moisture content of fresh pod pepper collected each time was calculated after being brought back to the laboratory for drying, and then the yield of dry pod pepper was calculated (Zhang et al., 2021a).

### 2.7 Calculations and statistical analysis

#### 2.7.1 Nutrient accumulation

The nutrient accumulation were calculated using the methods described by Ning et al. (2022).


NA=NC×DB

where NA stands for nutrient accumulation (kg·ha^-1^), NC stands for nutrient concentration (%), DB stands for dry biomass (kg·ha^-1^).

#### 2.7.2 Fertilizer utilization

The fertilizer utilization were calculated using the methods described by Zhang et al. (2022c).


$$\text{AE}=\frac{{\text{Y}}_{\text{F}}-{\text{Y}}_{\text{CK}}}{{\text{NI}}_{\text{F}}}$$

$$\text{RE}=\frac{{\text{NA}}_{\text{F}}-{\text{NA}}_{\text{CK}}}{{\text{NI}}_{\text{F}}}$$

where AE stands for agronomic efficiency (kg·kg^-1^), Y_F_ stands for dry yield of fertilization treatment (kg·ha^-1^), Y_CK_ stands for dry yield of CK treatment (kg·ha^-1^), RE stands for recovery efficiency (%), NA_F_ stands for nutrient accumulation of fertilization treatment (kg·ha^-1^), NA_CK_ stands for nutrient accumulation of CK treatment (kg·ha^-1^), and NI_F_ stands for nutrient input of fertilization treatment (kg·ha^-1^).

#### 2.7.3 Economic benefits

The economy benefits were calculated using the methods described by Zhang et al. (2022c).


OV=Y×UP

NEI=OV−FV

where OV stands for output value (RMB·ha^-1^), Y stands for yield of dry pod pepper (kg·ha^-1^), UP stands for unit price of dry pod pepper (RMB·kg^-1^), NEI stands for net income (RMB·ha^-1^), and FV stands for fertilizer value (RMB·ha^-1^). In the calculation of economic benefits, the unit price of dry pod pepper was 20.00 RMB·kg^-1^. The fertilizer of chemical fertilizer, biochar and vermicompost were 3500, 2000 and 1000 RMB·t^-1^, respectively.

### 2.8 Statistical analysis

The results are presented as mean ± standard error. The data were statistically analyzed using SPSS 18.0 (SPSS Inc., Chicago, IL, USA) by one-way ANOVA and Duncan’s method for multiple comparisons at *p*<0.05 significance level. The figures were conducted with Origin 8.0 (Origin Lab Corporation, USA).

## 3 Results

### 3.1 Effects of biochar and vermicompost application on yield of continuous cropping pepper

**Figure 2:**
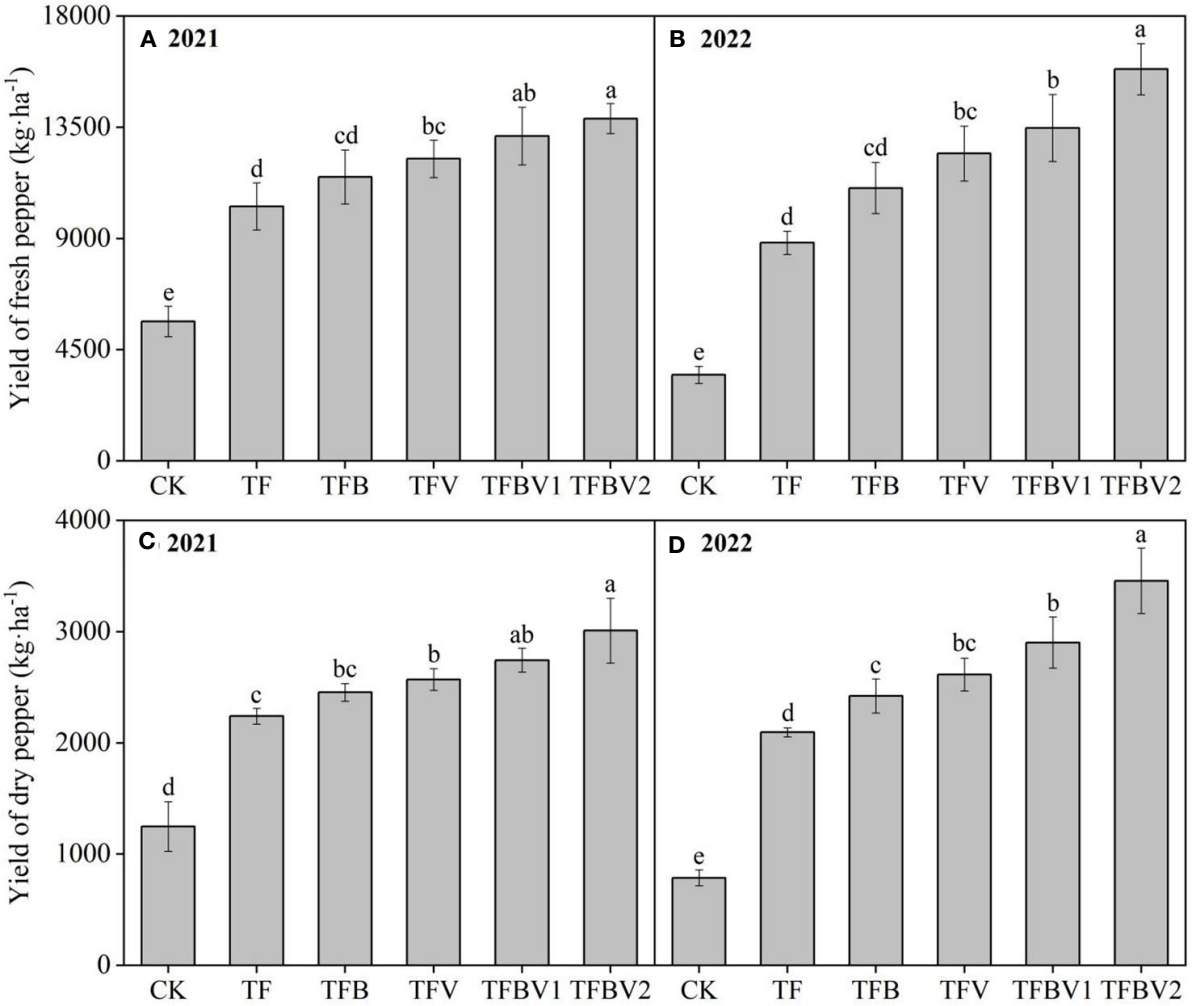
Effects of biochar and vermicompost application on yield of continues cropping pepper in 2021 **(A, C)** and 2022 **(B, D)**. CK - no fertilizer; TF - traditional fertilization of local farmers; TFB - TF combined with 3000 kg·ha^-1^ of biochar; TFV - TF combined with 3000 kg·ha^-1^ of vermicompost; TFBV1 - TF combined with 1500 kg·ha^-1^ of biochar and 1500 kg·ha^-1^ of vermicompost; TFBV2 - TF combined with 3000 kg·ha^-1^ of biochar and 3000 kg·ha^-1^ of vermicompost. Different lowercase letters indicate significant differences among different treatments at *p* <0.05 by the Duncan’s MRT method.

Biochar and vermicompost amendments had a positive effect on the yield of pod pepper (**Figure 2**). Compared with the TF treatment, the application of biochar and vermicompost alone or in combination (TFB, TFV, TFBV1, and TFBV2) increased the yield of fresh pod pepper by 24.38–50.03% (2021) and 31.61–88.92% (2022). TFBV2 treatment resulted in the highest yield of fresh pod pepper in two years, especially in 2022, with a maximum yield of 15853 kg·ha^-1^. It is noteworthy that the yield of fresh pod pepper in 2022 was reduced by 844 kg·ha^-1^ in the TF treatment compared with 2021, which implies that continuous cropping without organic amendment may cause yield reduction. In addition, the results showed that the change in dry pepper yield was similar to that in the fresh pepper yield. Compared with the TF treatment, the application of biochar and vermicompost alone or in combination significantly increased the yield of dry pod pepper by 14.69–40.63% and 21.44–73.29% in 2021 and 2022, respectively. The TFBV2 treatment showed the highest yield of dry pod pepper in the two years, which was 3009 kg·ha^-1^ in 2021 and 3457 kg·ha^-1^ in 2022.

### 3.2 Effects of biochar and vermicompost application on fruit quality of fresh pepper

The application of biochar and vermicompost ameliorated the quality of fresh pepper fruits, especially the reducing sugar, VC, and nitrate contents (**Table 2**). The results showed that the free amino acid content did not differ among all treatments in 2021 but was significantly higher in the TFBV1 and TFBV2 treatments than in the TF treatment in 2022. Compared with the TF treatment, the application of biochar and vermicompost alone or in combination increased the reducing sugar by 7.82–18.93% and 8.53–23.51% in 2021 and 2022, respectively. The reducing sugar content of the biochar and vermicompost combined application treatments (TFBV1 and TFBV2) increased by 5.80–10.30% in 2021 and 4.73–13.80% in 2022 compared with the application alone (TFB and TFV). Meanwhile, the application of biochar and vermicompost alone or in combination increased the VC content by 10.00–19.33% in 2021 and 18.49–30.14% in 2022. In addition, compared with the TF treatment, the application of biochar and vermicompost alone or in combination reduced the nitrate content of fresh fruits by 9.16–38.16% in 2021 and 12.99–40.23% in 2022, with the combination of biochar and vermicompost being the most effective.

**Table 2:** Effects of biochar and vermicompost application on quality of fresh pepper.

Year	Treatments	Free amino acid (g·kg^-1^)	Reducing sugar (mg·kg^-1^)	VC (g·kg^-1^)	Nitrate (mg·kg^-1^)
2021	CK	5.56 ± 0.16 a	39.98 ± 2.06 d	1.40 ± 0.03 c	78.81 ± 4.81 b
	TF	5.69 ± 0.09 a	46.18 ± 1.32 c	1.50 ± 0.12 bc	88.47 ± 3.92 a
	TFB	5.74 ± 0.30 a	49.79 ± 1.58 b	1.65 ± 0.11 ab	80.37 ± 5.50 b
	TFV	5.76 ± 0.49 a	50.00 ± 2.39 b	1.67 ± 0.13 ab	68.56 ± 3.25 c
	TFBV1	5.98 ± 0.18 a	52.90 ± 1.21 ab	1.72 ± 0.15 a	60.09 ± 2.24 d
	TFBV2	5.99 ± 0.11 a	54.92 ± 1.95 a	1.79 ± 0.08 a	54.71 ± 3.60 d
2022	CK	5.53 ± 0.13 b	39.02 ± 0.52 e	1.33 ± 0.06 c	71.98 ± 3.34 b
	TF	5.44 ± 0.48 b	46.06 ± 1.12 d	1.46 ± 0.06 c	80.29 ± 4.38 a
	TFB	5.74 ± 0.30 ab	49.99 ± 0.79 c	1.73 ± 0.14 b	69.86 ± 1.43 bc
	TFV	5.95 ± 0.17 ab	50.75 ± 0.85 c	1.77 ± 0.08 ab	66.14 ± 1.54 c
	TFBV1	6.11 ± 0.47 a	53.15 ± 0.99 b	1.84 ± 0.04 ab	54.58 ± 4.56 d
	TFBV2	6.26 ± 0.16 a	56.89 ± 1.64 a	1.90 ± 0.08 a	47.99 ± 1.71 e

Different lowercase letters in the same column indicate significant differences among different treatments at p< 0.05 level by the Duncan’s MRT method.

### 3.3 Effects of biochar and vermicompost application on nutrient accumulation in continuous cropping pepper

**Figure 3:**
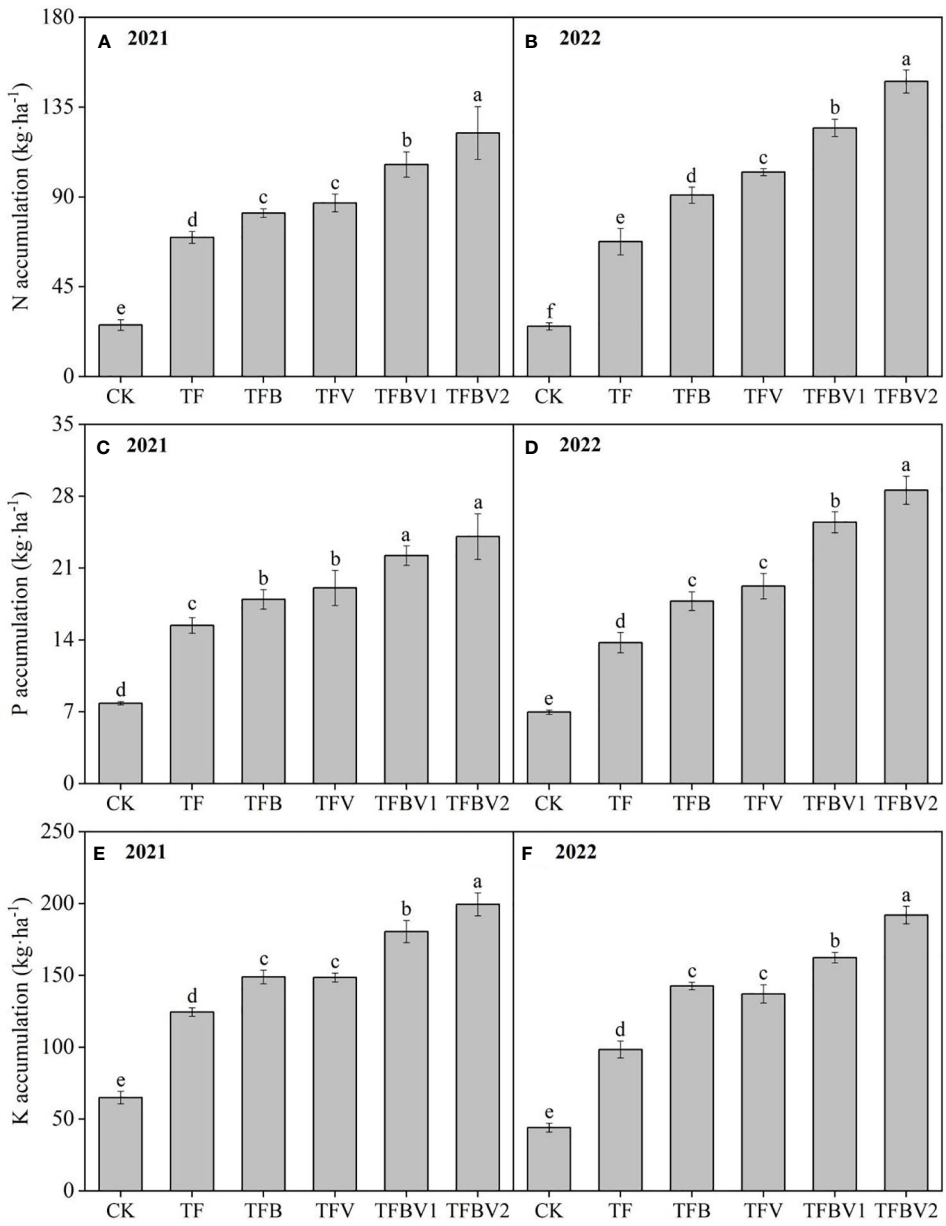
Effects of biochar and vermicompost application on nutrients accumulation in 2021 **(A, C, E)** and 2022 **(B, D, F)**. CK - no fertilizer; TF - traditional fertilization of local farmers; TFB - TF combined with 3000 kg·ha^-1^ of biochar; TFV - TF combined with 3000 kg·ha^-1^ of vermicompost; TFBV1 - TF combined with 1500 kg·ha^-1^ of biochar and 1500 kg·ha^-1^ of vermicompost; TFBV2 - TF combined with 3000 kg·ha^-1^ of biochar and 3000 kg·ha^-1^ of vermicompost. Different lowercase letters indicate significant differences among different treatments at *p* <0.05 by the Duncan’s MRT method.

The application of biochar and vermicompost positively affected the NPK nutrient accumulation (**Figure 3**). Compared with the TF treatment, the N, P, and K accumulation of biochar and vermicompost application alone or in combination increased by 17.49–75.10%, 16.38–56.11%, and 19.52–60.05% in 2021, whereas they increased by 34.36–118.67%, 29.36–107.92%, and 44.89–94.98% in 2022. The N, P, and K accumulation of biochar and vermicompost combined application treatments (TFBV1 and TFBV2) increased by 18.45–49.05%, 16.46–34.09%, and 21.15–34.22% in 2021 compared with the application alone (TFB and TFV), whereas they increased by 21.60–62.74%, 32.38–60.74%, and 13.81–39.98% in 2022.

### 3.4 Effects of biochar and vermicompost application on fertilizer utilization of continuous cropping pepper

**Table 3** shows that the application of biochar and vermicompost significantly increased fertilizer efficiency. Compared with the TF treatment, the AE_N_, AE_P_, and AE_K_ of biochar and vermicompost application alone or in combination increased by 35.15–97.58%, 35.39–97.67%, and 35.15–97.58% in 2021, whereas they increased by 35.27–120.76%, 35.37–120.92%, and 35.27–120.76% in 2022. The AE of the TFBV1 and TFBV2 treatments was higher than that of the TFB and TFV treatments, and the TFBV2 treatment showed the highest AE in both years. Similarly, compared with the TF treatment, the RE_N_, RE_P_, and RE_K_ of biochar and vermicompost application alone or in combination increased by 27.83–119.40%, 33.25–114.13%, and 40.18–125.43% in 2021, whereas they increased by 54.74–189.00%, 59.50–218.86%, and 71.14–172.03% in 2022, respectively. The combined biochar and vermicompost treatments (TFBV1 and TFBV2) showed average increases in RE_N_, RE_P_, and RE_K_ of 12.84, 7.31%, and 14.53%, respectively, compared with those applied alone (TFB and TFV).

**Table 3:** Effects of biochar and vermicompost application on fertilizer utilization of continues cropping pepper.

Year	Treatments	AE (kg·kg^-1^)			RE (%)		
		AE_N_	AE_P_	AE_K_	RE_N_	RE_P_	RE_K_
2021	CK	—	—	—	—	—	—
	TF	3.30 ± 1.21 c	9.89 ± 3.63 c	3.30 ± 1.21 c	16.24 ± 0.88 d	8.42 ± 0.93 c	22.10 ± 1.61 d
	TFB	4.46 ± 1.12 bc	13.39 ± 3.35 bc	4.46 ± 1.12 bc	20.76 ± 0.75 c	11.22 ± 1.23 bc	31.11 ± 0.37 c
	TFV	4.89 ± 0.59 abc	14.67 ± 1.78 abc	4.89 ± 0.59 abc	22.64 ± 2.37 c	12.48 ± 1.86 b	30.98 ± 2.63 c
	TFBV1	5.53 ± 0.43 ab	16.60 ± 1.28 ab	5.53 ± 0.43 ab	29.78 ± 1.42 b	15.98 ± 0.89 a	42.78 ± 1.93 b
	TFBV2	6.52 ± 1.60 a	19.55 ± 4.79 a	6.52 ± 1.60 a	35.63 ± 3.96 a	18.03 ± 2.51 a	49.82 ± 1.44 a
2022	CK	—	—	—	—	—	—
	TF	4.48 ± 0.55 d	13.43 ± 1.66 d	4.48 ± 0.55 d	15.73 ± 1.89 e	7.53 ± 1.10 d	20.13 ± 2.06 d
	TFB	6.06 ± 0.75 c	18.18 ± 2.24 c	6.06 ± 0.75 c	24.34 ± 2.01 d	12.01 ± 0.77 c	36.50 ± 1.32 c
	TFV	6.77 ± 0.80 bc	20.31 ± 2.41 bc	6.77 ± 0.80 bc	28.63 ± 1.24 c	13.64 ± 1.61 c	34.45 ± 1.73 c
	TFBV1	7.84 ± 0.94 b	23.51 ± 2.83 b	7.84 ± 0.94 b	36.83 ± 2.06 b	20.56 ± 1.27 b	43.80 ± 2.24 b
	TFBV2	9.89 ± 0.83 a	29.67 ± 2.49 a	9.89 ± 0.83 a	45.46 ± 1.85 a	24.01 ± 1.37 a	54.76 ± 2.14 a

AE_N_, AE_P_, and AE_K_ stand for agronomic efficiency of N, P, and K. RE_N_, RE_P_, and RE_K_ stand for recovery efficiency of N, P, and K. Different lowercase letters in the same column indicate significant differences among different treatments at p< 0.05 level by the Duncan’s MRT method.

### 3.5 Effects of biochar and vermicompost application on the economic benefits of continuous cropping pepper

**Table 4** shows that the application of biochar and vermicompost was economically beneficial for pod pepper. Compared with the TF treatment, the application of biochar and vermicompost alone or in combination increased the output value of dry pod pepper by 14.69–40.63% (2021) and 23.72–73.29% (2022), respectively. The output value of the TFBV2 treatment was the highest over two years. In addition, compared with the TF treatment, the net income of biochar and vermicompost alone or in combination increased by 0.77–22.34% in 2021 and 8.82–59.96% in 2022. The net income of the combined biochar and vermicompost application treatments (TFBV1 and TFBV2) increased by 4.57–21.40% in 2021 and 9.82–46.99% in 2022 compared to the application alone (TFB and TFV). The TFBV2 treatment showed the highest net income of dry pod pepper in the two years, at 45937 RMB·ha^-1^ in 2021 and 61814 RMB·ha^-1^ in 2022.

**Table 4:** Effects of biochar and vermicompost application on economic benefits of continues cropping pepper.

Year	Treatments	Output value (RMB·ha^-1^)	Fertilizer input (RMB·ha^-1^)	Net income (RMB·ha^-1^)
2021	CK	24990 ± 4461 d	—	24990 ± 4461 c
	TF	42799 ± 2102 c	5250	37549 ± 2102 b
	TFB	49088 ± 1577 b	11250	37838 ± 1577 b
	TFV	51403 ± 1951 b	8250	43153 ± 1951 ab
	TFBV1	54876 ± 2163 ab	9750	45126 ± 2163 a
	TFBV2	60187 ± 5847 a	14250	45937 ± 5847 a
2022	CK	17311 ± 1576 e	—	17311 ± 1576 e
	TF	43894 ± 2927 d	5250	38644 ± 2927 d
	TFB	53304 ± 3366 c	11250	42054 ± 3366 cd
	TFV	57518 ± 3229 bc	8250	49268 ± 3229 bc
	TFBV1	63857 ± 5054 b	9750	54107 ± 5054 b
	TFBV2	76064 ± 6452 a	14250	61814 ± 6452 a

Different lowercase letters in the same column indicate significant differences among different treatments at p< 0.05 level by the Duncan’s MRT method.

### 3.6 Effects of biochar and vermicompost application on soil fertility after pepper harvest

The physicochemical properties of the soil after harvesting peppers were shown in **Table 5**. Compared with TF treatment, biochar and vermicompost applied alone or in combination significantly increased soil pH (4.00-17.53%), SOM (11.55-87.30%), TN (17.57-68.22%), AP (26.30-137.55%), and AK (47.84-107.71%). In addition, the combined application of biochar and vermicompost was more effective in terms of soil fertility enhancement compared to biochar and vermicompost alone.

## 4 Discussion

As new type of organic amendment, biochar and vermicompost have been proven to have excellent effects on crop yield and soil improvement (Lee et al., 2018; Wang et al., 2018; Wang et al., 2019). The results of this study also confirmed the improvement effect of biochar and vermicompost on continuous cropping peppers in karst areas. The results of this study showed that the application of biochar and vermicompost, alone or in combination, had a positive effect on the yield of continuous cropping pepper in karst areas. Compared with the TF treatment, the yield of fresh pepper increased by 24.38–50.03% in 2021 and 31.61–88.92% in 2022, whereas the yield of dry pepper increased by 14.69–40.63% in 2021 and 21.44–73.29% in 2022. It has been shown that biochar can improve soil quality, which not only solves the problem of soil consolidation due to excessive fertilizer application but also achieves increased crop yield (Phares and Akaba, 2022; Yan et al., 2022; Wang et al., 2023). Biochar is rich in mineral elements and has a large specific surface area that can directly adsorb nutrients from the soil and reduce nutrient loss (Li et al., 2023; Lv et al., 2023). Furthermore, biochar application can stimulate the activity of soil microorganisms and improve the soil microbiological environment, which is beneficial for promoting crop root growth and yield (Manzoor et al., 2022; Wu et al., 2022; Wan et al., 2023). The positive effects of vermicompost on crop yields can be attributed to multiple factors. Studies have shown that vermicompost can not only activate enzyme activities involved in chlorophyll synthesis, plant growth, and fruit ripening but also increase the availability of available nutrients in the soil (Zuo et al., 2018; Liu et al., 2022). Some studies have confirmed that vermicompost also contains high amounts of cytokinins, growth hormones, and humic acids, which can promote crop development and yield (Arancon et al., 2010). Notably, the co-application of biochar and vermicompost in this study resulted in a better increase in yield than the application alone (with an average yield increase of 19.39% for fresh peppers and 20.38% for dry peppers), indicating that the co-application of biochar and vermicompost had a synergistic effect. This conclusion was also confirmed by previous studies, which noted that this could be related to the combined application of biochar and vermicompost to further improve the nutrient status and microbial environment of continuous crop barrier soils, particularly by inhibiting the growth of harmful pathogens (Wu et al., 2019; Wang et al., 2021; Rivelli and Libutti, 2022).

Improving the quality of agricultural products is the core of high-quality agricultural development. In this study, the application of biochar and vermicompost alone or in combination increased free amino acids, reducing sugars, and VC content in fresh pepper fruits and significantly reduced the nitrate content in fruits (**Table 2**), which indicated that organic remediation materials had a positive effect on fruit quality. Owing to the stable fertilization and continuous release of nutrients, biochar application facilitates the coordination and balance of crop nutrient metabolism, which contributes to the improvement in fruit quality (Almaroai and Eissa, 2020; Lévesque et al., 2020). In additionally, the improvement in fruit quality might be because the application of biochar increased the photosynthetic rate of leaves and facilitated the transport of photosynthetic products to the fruit, which is also beneficial for fruit quality (Akhtar et al., 2014; Zeeshan et al., 2020). Similarly, this positive effect can be explained by the fact that vermicompost is rich in essential amino acids such as glutamic acid and glycine, which can be involved in the soluble sugar metabolic process through plant roots and promote the enhancement of soluble sugar content in fruits (Wang et al., 2010; Scaglia et al., 2016). In this study, the application of biochar and vermicompost significantly reduced the nitrate content of pepper fruits, and the nitrate content of the TFBV1 and TFBV2 treatments was significantly lower than that of the TFB and TFV treatments. Biochar increases the adsorption of NH_4_^+^ and reduces the conversion of NH_4_^+^ to NO_3_^-^, which facilitates the reduction of nitrate content (Lou et al., 2015; Khajavi-Shojaei et al., 2023). However, the input of amino acid nitrogen into vermicompost can cause a significant decrease in the nitrate content of fruits through the inhibition of nitrate reductase, which may be responsible for the decrease in the nitrate content of fruits (Márquez-Quiroz et al., 2014; Doan et al., 2015). Interestingly, the synergistic benefits of biochar and vermicompost in improving the quality of continuous cropping pepper were also observed in this study, which may be related to the complementary effects of both biochar and vermicompost (Hafez et al., 2020; Ebrahimi et al., 2021; Wang et al., 2021). This may be because biochar and vermicompost can complement each other as vermicompost provides nutrients, and biochar increases the cation exchange capacity and C fixation in the long term (Álvarez et al., 2018).

Studies have shown that organic amendments are rich in nutrients and small organic molecules that are required for plant growth. These substances are easily converted into active components that can be absorbed and used by plants with the help of microorganisms, facilitating the accumulation of nutrients in plants (Singh et al., 2008; Wang et al., 2020). Organic amendments applied to soil can increase the activity of soil microorganisms and soil nutrient content, which is also beneficial for promoting nutrient decomposition and release, as well as plant uptake and utilization of nutrient elements (Srivastava et al., 2012; Kuppusamy et al., 2016; Phares and Akaba, 2022). In this study, the application of biochar and vermicompost alone or in combination not only significantly increased the accumulation of NPK (**Figure 3**) but also significantly improved the fertilizer utilization efficiency (**Table 3**). Studies have shown that biochar application increases the soil C/N ratio and limits nitrogen conversion and denitrification by soil microorganisms, facilitating the sequestration of NH_4_^+^ and NO_3_^-^ in the soil (Zhao et al., 2022; Ullah et al., 2023). In addition, biochar can be used as an alternative to traditional phosphorus fertilizers because of its inherently high effective phosphorus content, which can also alter the cycling and effectiveness of phosphorus in soils through the adsorption and desorption of phosphorus and regulation of the soil microbial community structure (Kong et al., 2023; Pokharel and Chang, 2023; Yan et al., 2023). Similarly, in addition to the direct contribution of potassium to biochar, biochar can also increase the effective soil potassium content and improve potassium fertilizer use efficiency by stimulating microbial activity (Xia et al., 2022; Yang et al., 2023). These views can also be confirmed by the soil fertility enhancement after pepper harvest in this study (**Table 5**). Notably, the addition of vermicompost may further enhance the soil amendment effect of biochar because of the richness of vermicompost in beneficial microorganisms, which can further activate and colonize the pore structure of biochar (Zhao et al., 2020; Wu et al., 2022). Furthermore, vermicompost contains beneficial microorganisms that can inhibit the growth of pathogenic fungi by inducing the secretion of antibacterial substances, including antibiotics and cell wall-degrading enzymes, particularly in soils with continuous cropping barriers (Szczech et al., 1993; Xiao et al., 2016).

**Table 5:** Effects of biochar and vermicompost application on soil fertility of after pepper harvest.

Treatments	pH	SOM (g·kg^-1^)	TN (g·kg^-1^)	AP (mg·kg^-1^)	AK (mg·kg^-1^)
CK	6.17 ± 0.10 cd	18.84 ± 2.13 c	1.34 ± 0.07 e	29.08 ± 2.39 f	122.17 ± 19.39 e
TF	6.11 ± 0.04 d	19.43 ± 2.51 c	1.57 ± 0.05 d	47.43 ± 2.92 e	197.89 ± 17.97 d
TFB	6.68 ± 0.05 b	28.54 ± 2.34 b	2.01 ± 0.04 b	59.90 ± 7.65 d	393.49 ± 21.20 ab
TFV	6.35 ± 0.15 c	21.67 ± 2.85 c	1.85 ± 0.09 c	73.32 ± 4.95 c	292.57 ± 15.43 c
TFBV1	6.71 ± 0.15 b	27.10 ± 2.39 b	2.12 ± 0.09 b	98.91 ± 2.59 b	369.92 ± 20.54 b
TFBV2	7.18 ± 0.12 a	36.39 ± 1.59 a	2.64 ± 0.09 a	112.66 ± 5.64 a	411.04 ± 28.48 a

Different lowercase letters in the same column indicate significant differences among different treatments at p< 0.05 level by the Duncan's MRT method.

The pursuit of greater economic benefits is the driving force behind farmers’ motivations to cultivate crops. In this study, the net income increased by 0.77–22.34% in 2021 and 8.82–59.96% in 2022 for biochar and vermicompost application alone or in combination (**Table 4**), and the net income effect was optimal for the co-application of biochar and vermicompost (TFBV1 and TFBV2). This indicates that although the input of biochar and vermicompost increased the production cost of pepper, it did not reduce the net income but rather contributed to economic efficiency. However, it is worth noting that although the output value of the TFV treatment increased by 4.72% (2021) and 7.91% (2022) compared with the TFB treatment under the same application rate conditions, the net income of the TFV treatment increased by 14.05% (2021) and 17.15% (2022) compared with the TFB treatment. This is because of the difference in fertilizer cost inputs, as biochar is twice the price of vermicompost, which also indicates that vermicompost may be a good application prospect as a low-cost and efficient soil amendment (Gebisa et al., 2021; Tascı and Kuzucu, 2023). Therefore, cheaper biochar materials or preparation technologies are key directions for future research, which may be more conducive to reducing the input cost of biochar in agricultural production and thus achieve better economic returns. Furthermore, organic amendments are mainly used to improve crop yield and quality by regulating the microecological environment of continuous cropping soils, and further research on the structure and function of soil microorganisms will be the focus of our future studies.

## 5 Conclusion

This study showed that the application of biochar and vermicompost, alone or in combination, was beneficial for increasing productivity and improving fruit quality, fertilizer utilization, and economic efficiency of continuous pepper cropping in karst areas of southwest China. Overall, the co-application of biochar and vermicompost was the most effective in improving the production capacity and economic benefits of the continuous cropping of pepper. Therefore, the combined application of biochar and vermicompost (3000 kg·ha^-1 +^ 3000 kg·ha^-1^) is recommended for the alleviation of continuous pepper cropping in karst yellow soil.

## Data availability statement

The original contributions presented in the study are included in the article/supplementary material. Further inquiries can be directed to the corresponding author.

## Author contributions

MZ and JG designed the study and wrote the manuscript. MZ, YL, QW and MW performed the experiments. MZ, LL, and XG interpreted the results of the experiments and edited and revised the manuscript. JG approved the final version of the manuscript. All authors contributed to the article and approved the submitted version.
